# Selection of Boar Sperm by Reproductive Biofluids as Chemoattractants

**DOI:** 10.3390/ani11010053

**Published:** 2020-12-30

**Authors:** Luis Alberto Vieira, Alessia Diana, Cristina Soriano-Úbeda, Carmen Matás

**Affiliations:** 1 Department of Physiology, Faculty of Veterinary Science, International Excellence Campus for Higher Education and Research Campus Mare Nostrum, University of Murcia, 30100 Murcia, Spain; tuncan24@yahoo.es (L.A.V.); alessia.diana@um.es (A.D.); cmsu1@um.es (C.S.-Ú.); 2 Institute for Biomedical Research of Murcia (IMIB-Arrixaca), 30120 Murcia, Spain; 3 Department of Veterinary and Animal Sciences, University of Massachusetts, Amherst, MA 01002, USA

**Keywords:** sperm selection, chemotaxis, reproductive fluids, IVF, IVC, porcine

## Abstract

**Simple Summary:**

Both in natural breeding and some assisted reproduction technologies, spermatozoa are deposited into the uterus. The journey the spermatozoa must take from the place of semen deposition to the fertilization site is long, hostile, and selective of the best spermatozoa. For the fertilization to succeed, spermatozoa are guided by chemical stimuli (chemoattractants) to the fertilization site, mainly secreted by the oocyte, cumulus cells, and other substances poured into the oviduct in the periovulatory period. This work studied some sources of chemotactic factors and their action on spermatozoa functionality in vitro, including the fertility. A special chemotactic chamber for spermatozoa selection was designed which consists of two wells communicated by a tube. The spermatozoa are deposited in well A, and the chemoattractants in well B. This study focuses on the use of follicular fluid (FF), periovulatory oviductal fluid (pOF), conditioned medium from the in vitro maturation of oocytes (CM), and progesterone (P4) as chemoattractants to sperm. The chemotactic potential of these substances is also investigated as related to their action on CatSper which is a calcium channel in the spermatozoa known to be sensitive to chemoattractants and essential for motility.

**Abstract:**

Chemotaxis is a spermatozoa guidance mechanism demonstrated in vitro in several mammalian species including porcine. This work focused on follicular fluid (FF), periovulatory oviductal fluid (pOF), the medium surrounding oocytes during in vitro maturation (conditioned medium; CM), progesterone (P4), and the combination of those biofluids (Σ) as chemotactic agents and modulators of spermatozoa fertility in vitro. A chemotaxis chamber was designed consisting of two independent wells, A and B, connected by a tube. The spermatozoa are deposited in well A, and the chemoattractants in well B. The concentrations of biofluids that attracted a higher proportion of spermatozoa to well B were 0.25% FF, 0.25% OF, 0.06% CM, 10 pM P4 and 0.25% of a combination of biofluids (Σ2), which attracted between 3.3 and 12.3% of spermatozoa (*p* < 0.05). The motility of spermatozoa recovered in well B was determined and the chemotactic potential when the sperm calcium channel CatSper was inhibited, which significantly reduced the % of spermatozoa attracted (*p* < 0.05). Regarding the in vitro fertility, the spermatozoa attracted by FF produced higher rates of penetration of oocytes and development of expanded blastocysts. In conclusion, porcine reproductive biofluids show an in vitro chemotactic effect on spermatozoa and modulate their fertilizing potential.

## 1. Introduction

Assisted reproductive technologies (ART) have been widely used in humans and farm animals in the last decades. Even though the female plays a critical role in the success of an ART program, we cannot ignore that the spermatozoa preparation has been essential to selecting the best and most capable spermatozoa population in an ejaculated sample [1], thus contributing to the increasing success of ART. However, the efficiency of many ART is still low, in part due to spermatozoa quality which directly influences fertilization and embryo development.

In standard methodologies of in vitro fertilization (IVF) in mammals, both male and female gametes are co-cultured without barriers regulating their interaction which causes a high number of spermatozoa to be simultaneously at the place of fertilization. Consequently, increased rates of polyspermy (more than one spermatozoon penetrating the oocyte) are achieved, compromising the embryo development. This is of particular interest to the porcine species, representing one of the major detrimental factors for an efficient and successful porcine IVF (reviewed by Romar et al. [2]).

There are several mechanisms within the female reproductive tract that regulate the number and quality of spermatozoa reaching the fertilization site to reduce the risk of polyspermy [3]. The in vitro simulation of the physico-chemical conditions in the oviduct at the time of fertilization can reduce the high incidence of polyspermy in porcine animals [4].

Under in vivo conditions, only progressively motile spermatozoa will move toward the oocytes. Current sperm selection assays based on sperm chemotaxis towards progesterone (P4) provide a sperm subpopulation enriched with spermatozoa that are capacitated, with intact DNA and low levels of oxidative stress [1]. Since the selected subpopulation of spermatozoa are in an optimum physiological state, it would be reasonable to suggest that the application of spermatozoa selection methods may improve the efficiency of the current ART. Despite the fact that P4 has been one of the most studied chemoattractant agents, other substances have also been shown to have this effect such as atrial natriuretic peptide (ANP), heparin, adrenaline, oxytocin, calcitonin, acetylcholine, and nitric oxide [5,6]. On the other hand, it has been suggested that estradiol (E2), cyclic AMP (cAMP) and cyclic GMP (cGMP) could be essential for chemotaxis because they increase the levels of intracellular calcium (Ca^2+^) in the sperm [7,8,9].

Villanueva-Diaz et al. [10], showed for the first time that crude human follicular fluid (FF) produced a chemical attraction for spermatozoa. FF increases the in vitro motility of spermatozoa especially since the original follicle is mature and stimulates capacitation and the acrosomal reaction of human sperm [11]. The presence of chemotactic agents has been shown in the FF of several species [5]. Therefore, FF facilitates spermatozoa reaching the fertilization site. Likewise, oviductal fluid (OF) and secretions of the cumulus cells (conditioned medium, CM) have been demonstrated to have chemotactic components [12,13,14,15].

In an in vitro chemotactic system, it is important to consider the optimum concentration at which a chemoattractant has an effect. A typical chemotaxis response is represented by a bell-shaped curve [12,13,14,16,17]. The present work analyzed the chemotactic effect of different biofluids (FF, OF, CM) and P4 individually or in combination on spermatozoa as well as their potential as sperm fertility modulators.

## 2. Material and Methods

### 2.1. Ethics Statement

The study was carried out in accordance with the Spanish Policy for Animal Protection RD 53/2013, which meets the European Union Directive 2010/63/UE on animal protection. All experimental protocols were approved by the Ethical Committee of Animal Experimentation of the University of Murcia and by the Animal Production Service of the Agriculture Department of the Region of Murcia (Spain) (ref. no. A13160609).

### 2.2. Reagents, Culture Media, and Solutions

All the chemicals used in this study were purchased from Sigma-Aldrich Química S.A. (Madrid, Spain) unless otherwise indicated. Tyrode’s albumin lactate pyruvate medium (TALP; [18]) was supplemented with 1.10 mmol/L sodium pyruvate and 3 mg/mL bovine serum albumin (BSA; A9647). Dulbecco’s PBS (DPBS) was supplemented with 1 mg/mL PVA and 0.005 mg/mL red phenol. North Carolina State University 37 medium (NCSU37; [19]) was supplemented with 0.57 mmol/L cysteine, 1 mmol/L dibutyryl cAMP, 5 µg/mL insulin, 50 μmol/L β-mercaptoethanol, 1 mmol/L glutamine, 10 IU/mL equine chorionic gonadotropin (eCG; Foligon; Intervet International BV, Boxmeer, Holland), 10 IU/mL human chorionic gonadotropin (hCG; Veterin Corion; Divasa Farmavic, Barcelona, Spain), and 10% (*v*/*v*) porcine FF [20]. A fixation solution of glutaraldehyde was prepared at 0.5% in phosphate-buffered saline (PBS). A staining solution of bisbenzimide (Hoechst 33342; Invitrogen, Thermo Fisher Scientific, Waltham, MA, USA, H1399) was prepared at 1% (*w*/*v*) in PBS. The P4 (P8783) solution was prepared in DMSO (D2650) at 1mg/mL and frozen at −20 °C until use. The solution of the CatSper channel inhibitor NNC 55-0396 (NNC; Cayman 17216) was prepared in DMSO at 8.8 mM and frozen at −20 °C until use.

### 2.3. Spermatozoa Collection and Preparation

Fresh ejaculated spermatozoa were obtained by the manual method from mature and tested fertility boars. Once in the laboratory, the spermatozoa were separated from the seminal plasma (SP) by centrifugation on a discontinuous density gradient (Percoll^®^, Pharmacia, Uppsala, Sweden) 45/90% (*v*/*v*) at 700× *g* for 30 min [21]. The pellet was resuspended in TALP medium previously equilibrated in a humified atmosphere of 5% CO_2_ in air at 38.5°C and centrifuged at 700× *g* for 5 min. Finally, the pellet of spermatozoa was resuspended in 5 mL of fresh TALP medium and the concentration of spermatozoa/mL was determined.

### 2.4. In Vitro Maturation (IVM) of Oocytes

Ovaries were obtained from prepuberal gilts at the local slaughterhouse and transported to the laboratory in saline solution at 38.5 °C within 1 h after the death of the animals. Cumulus oocyte complexes (COCs) were collected from antral follicles (3–6 mm diameter) and in vitro matured as described previously [4].

### 2.5. Follicular Fluid (FF), Periovulatory Oviductal Fluid (pOF) and Conditioned Medium (CM) Collection and Preparation

The FF was obtained making a pool by aspirating the liquid content of antral follicles (3–6 mm diameter) of prepuberal gilts ovaries, as described previously [22]. Periovulatory oviductal fluid (pOF) was obtained from a pool of porcine oviducts with ovaries close to ovulation according to the classification of Carrasco et al. [23]. The conditioned medium (CM), consisting of the pooled secretions of the cumulus cells, was obtained by pippeting NCSU37 medium from wells where groups of 50 COCs had completed the second phase of IVM (without dbAMPc, eCG and hCG) [4]. After collection, FF, pOF and CM were centrifugated at 7000× *g* and 4 °C for 10 min. After centrifugation, the supernatants were collected discarding the cellular debris and/or mucus at the bottom of the centrifuge tubes. All fluids, FF, pOF, and CM were aliquoted and frozen at −20 °C until use, avoiding repeated freezing-thawing cycles.

### 2.6. HPLC-MS Analysis

The separation and analysis of samples were performed with a HPLC-MS system consisting of an Agilent 1290 Infinity II Series HPLC (Agilent Technologies, Santa Clara, CA, USA) equipped with an Automated Multisampler module and a High-Speed Binary Pump and connected to an Agilent 6550 Q-TOF Mass Spectrometer (Agilent Technologies, Santa Clara, CA, USA) using an Agilent Jet Stream Dual electrospray (AJS-Dual ESI) interface.

Standards or samples (20 uL) were injected onto an Agilent Zorbax Eclipse Plus C18 (2.1 × 100 mm, 1.8 um) HPLC column, at a flow rate of 0.4 mL/min. The column was equilibrated at 40 °C. Solvents A (MilliQ water + 0.1% formic acid) and B (acetonitrile + 0.1% formic acid) were used for the compound separation with the following elution program: 2 min at 3% B, linear gradient from 3 to 100% B in 9 min, and 1 min at 100% B.

The mass spectrometer was operated in the positive mode. Extracted ion chromatograms of the following compounds were analyzed: 273.1849 > 255.1749 m/z for β-estradiol (E2), 315.2319 > 109.0660 for P4, 330.0566 > 136.0623 for AMPc and 346.0547 > 152.05686 for GMPc.

### 2.7. Sperm Chemotaxis System

For this study, a new chemotaxis chamber for spermatozoa was designed (Figure S1). It consisted of two 500 µL wells with stoppers connected by a tube of 250 µm in diameter and 850 µm in length. Spermatozoa suspension in TALP were added to well A in a final concentration of 20 × 10^6^ spermatozoa/mL. The chemoattractants under study were added to well B in TALP. This system was kept for 20 min in a humified atmosphere of 5% CO_2_ in air at 38.5 °C. After that incubation, the percentage of spermatozoa recovered in well B were determined.

### 2.8. Spermatozoa Motility

Spermatozoa motility was evaluated by computer assisted semen analysis (CASA) using the ISAS^®^ system (PROISER R + D S.L., Valencia, Spain), as protocolized in our laboratory [24]. The motion parameters determined into 3 different fields per sample were: the percentage of total motile spermatozoa (Mot, %), motile progressive spermatozoa (MotPro, %), curvilinear velocity (VCL, μm/s), straight line velocity (VSL, μm/s), average path velocity (VAP, μm/s), linearity of the curvilinear trajectory (LIN, ratio of VSL/VCL, %), straightness (STR, ratio of VSL/VAP, %), wobble of the curvilinear trajectory (WOB, ratio of VAP/VCL, %), amplitude of lateral head displacement (ALH, μm), and beat cross-frequency (BCF, Hz).

### 2.9. Spermatozoa Plasma Membrane Integrity

The plasma membrane integrity was analyzed by eosin-nigrosin staining as a reflection of spermatozoa viability, as described by Soriano-Úbeda et al. [24]. The percentage of membrane-intact spermatozoa (non-stained spermatozoa) were determined and considered viable spermatozoa.

### 2.10. In Vitro Fertilization (IVF) and Embryo Development (IVC)

The matured oocytes from IVM were mechanically denuded, washed in fresh TALP, and transferred in groups of 50 oocytes to 4-well plates (Nunc, Roskilde, Denmark) with 500 μL/well TALP. The insemination of oocytes was carried out giving a final concentration of 25 × 10^3^ spermatozoa/mL. The spermatozoa for insemination were those recovered in well B of the chemotactic system. Eighteen hours after insemination, putative zygotes were fixed in a 0.5% glutaraldehyde solution, stained in a bisbenzimide solution (Hoechst 33342) and examined under an epifluorescence microscopy (Leica^®^ DMR, USA), as it has been previously described [25]. The IVF parameters analyzed were the percentage of penetrated oocytes (Pen, %), percentage of monospermy of penetrated oocytes (Mon, %), mean number of spermatozoa per penetrated oocyte (Spz/O) and mean number of spermatozoa bound to zona pellucida (Spz/ZP). For the embryo development assessment, 18 h after insemination, putative zygotes were transferred to a culture dish for 7 days (168 h), as previously described [20]. For the first 48 h, the media used for IVC was NCSU23 [19]. At 48 h after the addition of spermatozoa to the chemotactic system, the cleavage was assessed under the stereomicroscope. On day 7 (168 h after the addition of spermatozoa to the chemotactic system), the blastocyst stage of development was assessed under the stereomicroscope and classified as in Bó and Mapletoft [26]. Blastocysts and expanded blastocysts were fixed and stained as described for putative zygotes, and the mean number of blastomeres was determined under an epifluorescence microscope (Leica^®^ DMR, Richmond, IL, USA).

### 2.11. Experimental Design

**Figure 1:**
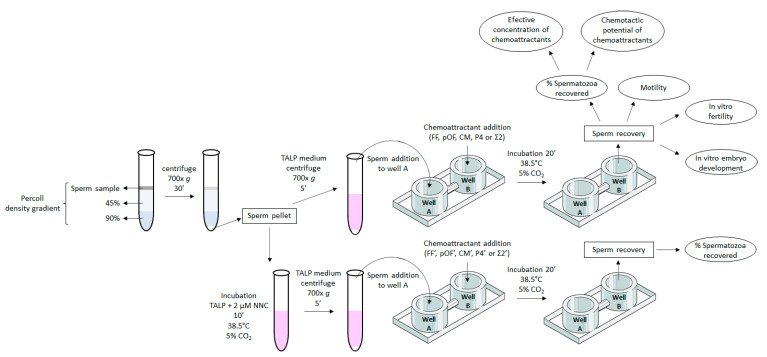
Diagram of the experimental design. Boar ejaculated spermatozoa were separated from seminal plasma by centrifugation on a discontinuous density gradient (Percoll). The pellet was resuspended in TALP medium supplemented or not with 2 µM of the CatSper inhibitor NNC 55-0396 (NNC). Spermatozoa resuspended in TALP + NNC were incubated for 10 min (38.5 °C, 5% CO_2_). Chemotactic chamber was loaded with 500 µL TALP medium per well. Chemoattractants were added to well B of the chemotactic chamber (in different concentrations depending on the experiment). Sperm samples in fresh TALP were centrifuged 700× *g*, 5 min and added to the well A of the chamber. The chemotactic system was incubated for 20 min (38.5 °C, 5% CO_2_). Spermatozoa recovered in well B were analyzed: % of spermatozoa recovered, motility, in vitro fertility, and in vitro development of blastocyst [just for spermatozoa selected by follicular fluid (FF)]. With the % of spermatozoa recovered in well B, it was also determined the effective concentration of chemoattractants (lowest concentration of chemoattractant that attracted the highest proportion of spermatozoa), and the chemotactic potential of chemoattractants [comparison of the most effective concentration of chemoattractants through the % of spermatozoa recovered in well B attracted by each chemoattractant and a combination of them (Σ)].

The present study investigated the chemotactic ability of the female reproductive fluids (FF, pOF, CM) and P4 on the selection of porcine spermatozoa and the fertility of those selected spermatozoa (Figure 1). Firstly, the characterization in content of E2, P4, cAMP, and cGMP in each pool of biofluids used in the present work was carried out. The concentration of those components was measured twice by HPLC-MS in the pools of FF, pOF and CM, and the results are shown in Table 1. Once characterized, the same pools of biofluids were used in the two experiments of this work.

#### Experiment 1. Spermatozoa selection by FF, pOF, CM, and P4

(1.1) Effective concentration of chemoattractants. Increasing concentrations of the chemoattractants (FF at 0.13, 0.25, 0.50, 1.00 and 1.50%; pOF at 0.13, 0.25, 0.50, 1.00 and 1.50%; CM at 0.03, 0.06, 0.13, 0.25 and 0.50%; P4 at 1.00, 2.50, 5.00, 7.50 and 10.00 pM) were added to well B of the chemotactic system. One experimental group in which there was no supplementation with any chemoattractant was included as control (C). Spermatozoa were added to the well A and incubated for 20 min. The percentage of spermatozoa recovered in well B at the end of the incubation period was determined, and the most effective concentration of each chemoattractant was established as the lowest concentration that attracted the highest proportion of spermatozoa. Four replicates were performed.

(1.2) Chemotactic potential of chemoattractants. Since all the chemoattractants coexist in combination in the female reproductive tract during the periovulatory stage, this work compared the effective concentration of the chemoattractants (FF, pOF, CM, and P4) and their possible synergy. For this purpose, a combination of the most effective concentrations of FF, pOF and CM were studied as the summation of the most effective concentrations of chemoattractants obtained in experiment 1.1 (Σ1 = 0.25% FF + 0.25% pOF + 0.06% CM). Due to the possible saturation of receptors [27], a second Σ group (Σ2) was included in which the total proportion of chemoattractants was limited to 0.25%. The proportion of each chemoattractant was maintained at 1/3 of the total 0.25% of biofluids in the chemotactic system (Σ2 = 1/3 of 0.25% FF + 1/3 of 0.25% OF + 1/3 of 0.06% CM). The chemotactic potential of chemoattractants and their combination was compared. A group in which no chemoattractants were added to well B was included as a control (C). Four replicates were performed.

(1.3) Motility of spermatozoa selected by chemoattractants. The possible influence of chemoattractants in motility of spermatozoa was studied in those spermatozoa selected in the chemotactic system by the chemoattractants FF, pOF, CM, P4 and Σ2. A group in which no chemoattractants were added to well B was included as a control (C). Four replicates were performed.

(1.4) Mechanism of action of chemoattractants. The P4 triggering of Ca^2+^ uptake into the spermatozoa takes place through CatSper [28] and seems to play a key role in chemotaxis [14,29,30,31,32]. To elucidate P4 dependence on the chemotactic potential, spermatozoa selected by the most effective concentration of each chemoattractant (FF, pOF, CM, P4) were compared to the selected spermatozoa preincubated for 10 min with 2 µΜ of the CatSper inhibitor NNC 55-0396 [28] (FF′, pOF′, CM′, P4′). The most effective combination of chemoattractants (Σ2 and Σ2′) were included in this study, and groups without chemoattractant supplementation in well B were included as controls (C and C′). Four replicates were performed. Additionally, the possible collateral effect of NNC on spermatozoa motility by CASA and the membrane integrity was evaluated in three replicates. For the membrane integrity study, 200 spermatozoa per experimental group and replicate were analyzed.

#### Experiment 2. In vitro fertility of spermatozoa selected by chemotaxis and in vitro development of embryos.

To study the functionality of the selected spermatozoa, IVF was performed with those spermatozoa recovered in well B of the chemotactic chamber. The experimental groups were established according to the chemoattractant used for sperm selection: 0.25% FF, 0.25% pOF, 0.06% CM, 10.00 pM P4, and Σ2. One group in which there was no supplementation with any chemoattractant in well B was included as control (C). A total of 702 oocytes in four replicates were inseminated, and they were fixed at 18 h post-insemination for IVF parameter evaluation. The experimental group with higher penetration in IVF, the FF, was tested also in IVC. For that purpose, a total of 1,520 oocytes in six replicates were inseminated and 18 h post-insemination they were transferred to NCSU23 medium for up to 7 days of culture.

### 2.12. Statistical Analysis

The statistical analyses were performed using SPSS v.20 (SPSS Inc. Chicago, IL, USA). The variables were analyzed by analysis of variance (ANOVA). When ANOVA revealed a significant effect, values were compared by the least significant difference pairwise multiple comparison Tukey post hoc test. The results were expressed as the mean ± standard error of the mean (SEM) and *p* < 0.05 was established to indicate statistical significance.

## 3. Results

### 3.1. Concentration of E2, P4, cAMP and cGMP in FF, pOF and CM

Despite chemotaxis being mainly attributed to P4, other components have been suggested as potential chemoattractants in the porcine species. This work characterized the pools of biofluids used in all experiments through the concentration of some molecules responsible for chemotaxis in some reproductive biofluids (Table 1). FF showed the highest amount of E2, P4, and cGMP, followed by pOF which showed the highest amount of cAMP. CM showed the lowest cAMP values but higher than pOF for E2 and P4. The concentration of cGMP was higher in FF than in E2, P4, and cAMP. cGMP represented the main component in pOF, also, followed by cAMP, P4, and E2. On the other hand, CM was richer in E2 than in the rest of the components analyzed. Interestingly, P4 was the third highest component in concentration of each biofluid analyzed.

### 3.2. Effective Concentration of Chemoattractants Selecting Spermatozoa

**Figure 2:**
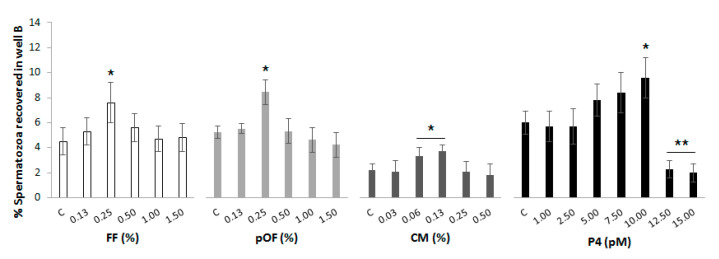
Effective concentration of chemoattractants. Increasing concentrations of the chemoattractants were added to well B of the chemotactic system. A group in which there was not a supplementation of any chemoattractant in well B was included as control (C) for each chemoattractant. Spermatozoa were added to well A in a concentration of 20 × 10^6^ spermatozoa/mL. The system was incubated for 20 min, 38 °C, 5% CO_2_ and 95% humidity. The results are expressed as the percentage of spermatozoa recovered in well B. The most effective concentration of each chemoattractant was established as the lowest concentration that attracted the highest proportion of spermatozoa. Four replicates were performed. Asterisks (*, **) indicate statistical differences between groups within the same chemoattractant (*p* < 0.05).

High concentrations of chemoattractants can saturate the cognate receptors and consequently the chemotactic response decreases. The results of the percentage of spermatozoa recovered in well B from those initially added to well A (called most effective concentration) for each chemoattractant are shown in Figure 2. The most effective concentration (*p* < 0.05) of each chemoattractant was: 0.25% FF (7.6 ± 1.6%), 0.25% pOF (8.4 ± 1.0%), 0.06% CM (3.3 ± 0.7%), 10.00 pM P4 (9.6 ± 1.6%).

### 3.3. Chemotactic Potential of Chemoattractants

The possible synergies between chemoattractants on spermatozoa are shown in Figure S2. The combination of a lower concentration of each chemoattractant (FF, pOF, CM) to a final concentration of 0.25% (Σ2) attracted a higher percentage of spermatozoa (Σ2 = 12.3 ± 1.3%; *p* < 0.05) than Σ1 (Σ1 = 8.0 ± 2.1%) and C (C = 8.9 ± 1.8%).

**Figure 3:**
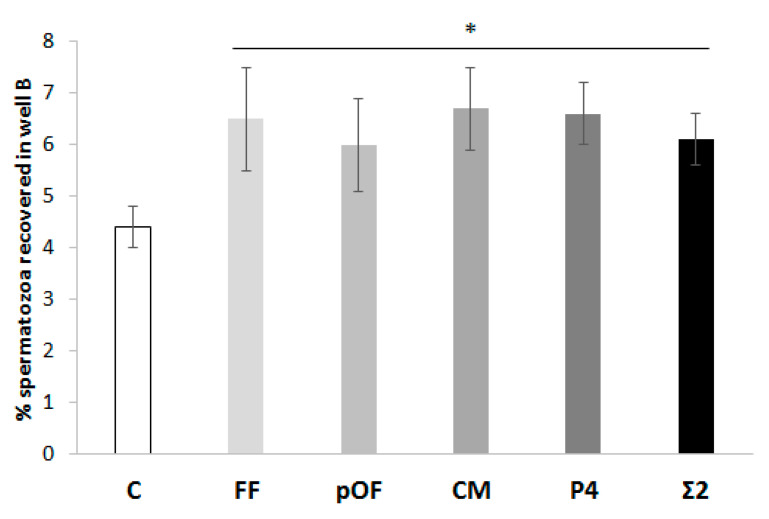
Chemotactic potential of chemoattractants. The percentage of spermatozoa attracted by the most effective concentration of each chemoattractant (0.25% FF, 0.25% pOF, 0.06% CM, 10.00 pM P4) was determined. The combination of the most effective concentration of chemoattractants (Σ2) according to experiment 1.1 was included in this study. A group in which there was not a supplementation of any chemoattractant in well B was included as control (C). The results are expressed as man ± SEM. Four replicates were performed. The asterisk (*) indicates significant statistical differences (*p* < 0.05).

The results of the chemotactic potential of chemoattractants are shown in Figure 3. The % of spermatozoa recovered when a chemoattractant (FF: 6.5 ± 1.0%; pOF: 6.0 ± 0.9%; CM: 6.7 ± 0.8%; P4: 6.6 ± 0.6%; Σ2: 6.1 ± 0.5%) was added to well B was higher (*p* < 0.05) than the control (C: 4.4 ± 0.4%).

### 3.4. Motility of Spermatozoa Selected by Chemoattractants

The motility of spermatozoa selected by the effective concentration of each chemoattractant is shown in Table 2. No statistical differences were found for any of the CASA parameters (*p* > 0.05).

### 3.5. Mechanism of Action of Chemoattractants through CatSper

**Figure 4:**
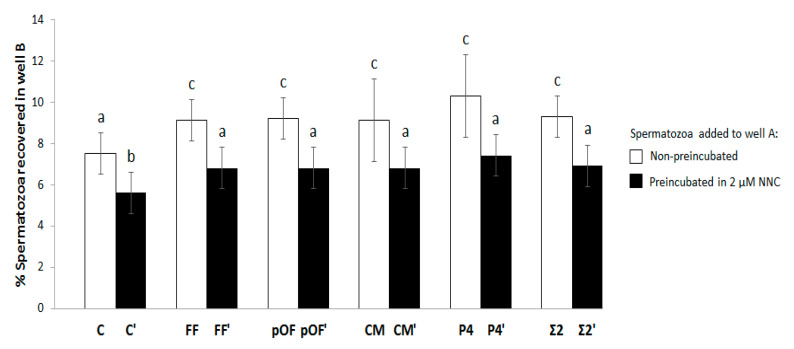
Chemotactic potential of chemoattractants and mechanism of action through CatSper. The percentage of spermatozoa attracted by the most effective concentration of each chemoattractant (0.25% FF, 0.25% pOF, 0.06% CM, 10.00 pM P4) was determined. That parameter was compared Table 10. min with 2 µΜ of the CatSper inhibitor NNC 55-0396 [28] (FF′, pOF′, CM′, P4′). The combination of the most effective concentration of chemoattractants according to experiment 1.1 (Figure S2; Σ2 and Σ2′) was included in this study. The groups in which there was not a supplementation of chemoattractant in well B for selecting spermatozoa were included as control (C and C′). The results are expressed as man ± SEM. Four replicates were performed. Different letters (a–c) indicate statistical differences between groups (*p* < 0.05).

Figure 4 shows the results of the concentrations of biofluids with higher chemotactic activity and it also shows that the chemotactic activity is related to CatSper. There was no difference in the % of spermatozoa attracted between chemoattractants or their combination (FF, pOF, CM, P4, Σ2; *p* > 0.05), or even when the spermatozoa were previously incubated with NNC (FF′, pOF′, CM′, P4′, Σ2′; *p* > 0.05). However, in both cases the % spermatozoa attracted were higher than their respective controls C and C′. The chemotactic action of all chemoattractants through CatSper was demonstrated by the reduction of the % of spermatozoa attracted by all chemoattractants until the point of being statistically similar to the control C. In other words, all chemoattractants in which spermatozoa had CatSper inhibited by NNC attracted the same % of spermatozoa as in the absence of chemoattractants. When comparing the effect of NNC on the controls (without chemoattractants), in C′ a significantly lower proportion of spermatozoa were recovered in well B as compared to C (*p* < 0.05). Additionally, Table S1 and Figure S3 showed that the motility and membrane integrity of spermatozoa is not affected by NNC.

### 3.6. Spermatozoa Selected by Chemotaxis to the Follicular Fluid (FF) Increases the Penetration of Oocytes In Vitro and the Rate and Quality of Blastocysts Production

During in vivo fertilization in the oviduct, the sperm must be guided towards the oocyte by different mechanisms, one of them is chemotaxis. Female reproductive biofluids can guide the spermatozoa and probably modulate their functionality, fertility, and potential in forming viable embryos. Table 3 shows the results of IVF with spermatozoa selected by chemotaxis. Spermatozoa selected by FF produced a higher % Pen (*p* < 0.05) but the same % Mon (*p* > 0.05) when compared to the rest of chemoattractants. In addition, spermatozoa selected by FF produced the highest proportion of spermatozoa bound to ZP (Spz/ZP), which was statistically the same to that obtained in C (*p* > 0.05). On the contrary, the spermatozoa selected by pOF and Σ2 showed the lowest % Pen, and Σ2 also produced the lowest Spz/ZP (*p* < 0.05).

Since the chemoattractant with the higher % Pen in IVF was FF, this group was also tested for IVC and the results are shown in Table 4. Although spermatozoa selected by FF did not modify the % of cleaved putative zygotes (2-cells; *p* > 0.05), they produced a significantly higher (*p* < 0.05) % of blastocysts (38.5 ± 3.7%) and expanded blastocyst (36.0 ± 2.5%) compared to the control group (27.6 ± 3.8% and 27.0 ± 2.2%, respectively). Furthermore, the number of cells of those blastocysts and expanded blastocyst were also significantly higher (*p* < 0.05) in the FF group (40.4 ± 2.3 and 54.5 ± 2.4, respectively) than in the control (35.9 ± 2.4 and 49.6 ± 2.1, respectively).

## 4. Discussion

After ovulation, spermatozoa that remain attached to the oviductal epithelial cells in the sperm reservoir are released and continue their journey to meet the oocyte. Since the oviduct is a very narrow and full of crypts duct, the process of gametes encountering cannot be random. Not all sperm will reach the oocyte, and a strict and selective process occurs during their path. It has been suggested that spermatozoa could be attracted by chemical factors that would be released by the oocyte, cumulus cells, and/or the epithelial oviductal cells. Some possible chemoattractants that facilitate spermatozoon-oocyte interaction are substances present in FF, OF [12,33], and other secretions surrounding the oocyte [12,13,34]. Chemotaxis has been described as a concentration-dependent [27] and species-specific phenomenon [12,13,14,17]. However, the chemical nature of chemoattractants, their receptors and the underlying signaling pathways are still a subject up for debate. In the porcine species, the main sperm-chemotactic role was attributed to P4 [14,32,35,36], but other components were also suggested as potential chemoattractants [37,38].

A typical chemotaxis response is represented by a bell curve, where at lower or higher attractant dilutions no chemotactic response is observed. However at intermediate attractant dilutions, the proportion of chemotactic cells is the greatest [39]. The chemoattractant concentrations obtained in this study were higher than those described in the literature for other species [12,13,17,40]. These differences can be attributed, on the one hand, to the working conditions, since the devices and volumes used by those authors are different compared to ours. On the other hand, those differences could be attributed to some molecular species-specific events during sperm capacitation. However, this last assumption cannot be corroborated with other studies since, to the best of our knowledge, there are not any published studies developed in the porcine species. Regarding the concentration of P4, there is an in-depth study, not only concerning its chemotactic activity [1] but also its participation in the activation of calcium channels involved in the hyperactive movement of the spermatozoa. The concentration of P4 that attracted the largest number of porcine spermatozoa to well B was 10.00 pM, and above this amount there was no greater response. Similar results were observed in human [36], rabbit [36] and mouse sperm [41].

Regarding the chemotactic potential, we did not find statistical differences between experimental groups, but the phenomenon of spermatozoa attraction was clear since there was a significantly lower % of spermatozoa attracted in their absence. This result was unexpected since, despite all biofluids having common components, the concentrations of those components are different. Thus, it would be reasonable to think that the chemoattraction potential can vary between biofluids. It is possible that the effective concentrations of chemoattractants in the biofluids used in the present work were too high. In that case, the saturation of receptors could be a plausible explanation for the lack of effect of the biofluids. Thus, the reduction in the concentration of these biofluids to elucidate their chemotactic potential would make sense. To our knowledge, there are not any studies that show the physiological concentration of chemoattractants in porcine reproductive biofluids. And those may also vary according to the different phase of the estrous cycle. In mice, Oliveira et al. [12] observed that pOF induced a high proportion of spermatozoa travelling longer distances toward the chemotactic gradient, while FF caused an increase in spermatozoa velocity. However, the reasons why the values of spermatozoa attraction obtained in this work with pOF were lower than those obtained with FF remains to be clarified.

It seems logical to think that a chemoattractant whose composition is closest to that found by the spermatozoa in vivo should be the one that attracts more spermatozoa. That is represented in this work as a combination of biofluids (Σ). Nevertheless, the results in this study did not show that. Considering that the in vivo contact of the sperm with chemoattractants occurs sequentially, one hypothesis could be that some receptors may be activated at a given moment and consequently induce the activation of other receptors later [13,36]. The exposure of spermatozoa to chemoattractants in this study was not sequential and the chemotactic receptors could have been activated at the same time preventing a greater chemotactic response.

Although Armon and Eisenbach [42] provided evidence that hyperactivation is part of the chemotactic response of the spermatozoa, the motility results found here by CASA showed no differences between the experimental groups. The results in other studies carried until now about whether motility is affected by chemotaxis are quite controversial. Other authors such as Fabbri et al. [43] observed, in humans, an increase in motility and hyperactivation, but Isobe et al. [44] did not observe variations of motility derived from chemotaxis. The main differences of the present study with previous studies can be that the sperm motility was analyzed after sperm migration towards the chemoattractant. Armon and Eisenbach [42] analyzed the sperm trajectory of swimming in a spatial chemoattractant gradient and observed that hyperactivation was significantly reduced by chemoattractants compared to controls. These authors suggested that with the increase in the chemoattractant concentration the capacitated sperm represses the hyperactivation but keeps swimming in favor of the concentration gradient of chemoattractant.

The importance of increased intracellular Ca^2+^ in spermatozoa has been demonstrated during chemotaxis [45]. In the chemotactic process the hyperactive movement is produced (regulated by the activation of CatSper channels) and the directionality of the spermatozoa towards the chemoattractant changes [29]. It has been shown in porcine species that the CatSper inhibitor NNC blocked the spermatozoa release from oviduct isthmic epithelial cells [46]. This study used the same concentration of NNC and showed a decrease in the percentage of spermatozoa that migrated to the chemoattractant in all groups, including the control. However, the sperm accumulation in well B appeared not to be completely abolished by NNC, since that reduction was also observed when no chemoattractant was added to the system. Other substances with chemotactic activity could be present in the biofluids. Some candidates could be the 8.6 kDa protein similar to apolipoprotein B2 [38], or maybe the antithrombin III [37] which is responsible for this migration when CatSper are inhibited. However, the mechanism by which these substances stimulate motility has not yet been elucidated. The inhibitor NNC is quite unspecific for CatSper, which means that it could be unsuitable for chemotaxis assays. Rennhack et al. [47] showed that NNC exhibit serious adverse reactions in human sperm and evoke a sizeable and sustained increase of [Ca^2+^]_i_ and pH_i_ [48,49,50] in addition to stimulating the acrosomal exocytosis [50].

Although P4 has been identified as the main chemoattractant in pOF, FF, and CM, other components with chemotactic activity are due to be present in biofluids. Therefore, we decided to determine the concentration of E2, P4, cAMP, and cGMP in the biofluids studied. The FF showed the highest concentrations of all of them except for cAMP. Interestingly, the FF was not the biofluid that showed the highest chemoattraction power. Perhaps some components offset the effect of others found in lower concentrations. For instance, cells are less sensitive to cGMP than to cAMP, but cGMP produces a more intense response [51]. Biofluids exhibit complex activity and the spermatozoa are simultaneously exposed to multiple ligands. This can lead to multiple effects and/or separate interactions. In this sense, the estrogen pretreatment elevates Ca^2+^ in spermatozoa apparently by a CatSper independent mechanism [28,52]. The estrogen pretreatment also reduces the concentration of Ca^2+^ in response to the stimulation with P4. However, that inhibition could be not produced when the concentrations of P4 are high [53].

Other questions that still need to be solved include why second messengers, such as cAMP and cGMP, are present in biofluids and their roles in chemotaxis. Purinergic signaling has been found to be a key component in the physiology of several tissues. Through the self-production of nucleotides and nucleosides and their binding to specific receptors, a wide range of cellular responses are modulated such as cell growth, differentiation, and motility. A clear example of the importance of these nucleotides was shown by Osycka-Salut et al. [54]. These authors demonstrated that bicarbonate in the extracellular medium produced an early increase in cAMP dependent on the soluble adenylyl cyclase (sAC) in the intra- and extracellular space in spermatozoa. Thus, it suggests that if the existence of a cAMP flow from the spermatozoa to the extracellular space were blocked, that it could result in the inhibition of capacitation. Another possible function of these nucleotides could be as an indicator of the direction to which the spermatozoon must go to find the oocyte, as in the case of sea urchins [55].

The functionality of the spermatozoa selected by pOF, FF, CM, and P4 was analyzed through IVF. The highest oocyte penetration was achieved when FF was used as the chemoattractant but the number of spermatozoa per oocyte was not affected by the biofluids used. A possible explanation of this would be that the FF increases the penetration of oocytes by modulating capacitation and acrosome reaction (reviewed by Hong et al. [11]) which consequently increases the penetration of oocytes. It would be logical to think that the number of spermatozoa per oocyte should also increase [56]. In this sense, Funahashi and Day [57] observed that the pre-fertilization incubation of porcine spermatozoa in suitable concentrations of porcine FF effectively reduces the incidence of polyspermy. Their results indicated that polyspermy blocking is produced by an interaction between FF and spermatozoa and not with oocytes. Another plausible explanation would be that the FF was the attractant that selects the subpopulation of spermatozoa with the highest quality and, therefore, with the highest penetration rate. These hypotheses are supported by the results of Ralt et al. [40], who reported that the FF potential to attract human spermatozoa is strongly correlated with the potential of those spermatozoa to fertilize the oocyte. However, under in vivo conditions, the spermatozoon contacts with other biofluids and biomolecules while it is attracted to the oocyte.

The lowest penetration rate obtained when spermatozoa were selected by pOF or Σ could be attributed to the presence of some factors that prevent capacitation before the spermatozoa contact with the oocyte. Soriano-Úbeda et al. [4] showed that pOF and CM decreases spermatozoa protein kinase A substrates and that tyrosine residues phosphorylation. And later, Zapata-Carmona et al. [25] showed that this process is reversible. During capacitation, the decapacitation factors from the SP are lost and that the intracellular Ca^2+^ concentration rises [58]. After that, the spermatozoa acquire additional PMCA4 from the oviduct via exosomes [59] which provide an adequate Ca^2+^ efflux to promote spermatozoa viability and prevent a premature acrosome reaction. Therefore, it cannot be discarded that some factors of pOF decreased spermatozoa capacitation, and other factors produced by the oocyte attracted the spermatozoa and prepared it for fertilization [60].

Until a few years ago, the research on embryo quality focused mainly on evaluating the impact of maternal factors on the embryo. However, it has recently been determined that paternal factors also share responsibility for contributing to better embryo quality. In our work, we have analyzed the quality of embryos obtained with spermatozoa selected by a density gradient and then migrated towards the FF. The results showed that there were no differences in the percentage of embryos divided at 48 h. However, those differences appeared later at the blastocysts and expanded blastocyst stages as well as in the number of cells that form those blastocysts. In both cases, the best quality was higher for the group of spermatozoa that migrated towards the FF. Therefore, the FF somehow selected the best spermatozoon. Gatica et al. [1] obtained better quality embryos when using sperm selected by chemotaxis and argued that this was due to less DNA damage that usually is very low in AI doses. In a previous study (data not shown) we analyzed DNA damage using acridine orange and the Halomax^®^ kit and observed that the % altered DNA was very close to 0%.

It is interesting to observe that the differences between the groups appear at 168 h (7 days) of embryo culture in the blastocyst stage. This fact leads us to think that not only selection by FF is important to obtain spermatozoa of higher quality, but this fluid can provide the spermatozoa with certain factors that improve subsequent embryonic development. Based on this data, we can hypothesize that the results obtained here could be regulated by the extracellular vesicles (EVs) present in the FF. It has been observed that a brief co-incubation is sufficient for transferring components from EVs to the spermatozoa. These EVs participate in this way in the sperm formation process while providing them different types of RNA [61]. All this could explain the improvement in the quality of the embryos obtained with spermatozoa selected by chemotaxis towards the FF [62]. In this work we have observed that the speed of the blastocysts to expand is greater when the spermatozoa are selected by FF. Supporting this theory, Fatehi et al. [63] reported that bovine embryos in the faster division stage are more likely to become blastocysts, and that embryos of rapid division are associated with higher gestation rates.

## 5. Conclusions

Under in vitro conditions, the FF selects the best spermatozoa for an optimum and more physiological interaction with the oocyte. The knowledge acquired with this work can be useful in improving the current ART performed in animal production and as model for human clinic assays. From this point on, more studies are necessary to deepen the knowledge of gamete interaction in the physiological environment.

## Figures and Tables

**Table 1:** Concentration of β-estradiol (E2), progesterone (P4), 3′,5′-cyclic adenosine monophosphate (cAMP), and guanosine 3′-5′-cyclic monophosphate (cGMP) in reproductive biofluids used as chemoattractants. HPLC-MS was performed twice in each pool of biofluids.

	FF (ng/mL)	pOF (ng/mL)	CM (ng/mL)
**E2**	0.71 ± 0.60	<0.01 ± 0.00	0.10 ± 0.10
**P4**	0.53 ± 0.01	0.05 ± 0.04	0.08 ± 0.05
**cAMP**	0.11 ± 0.04	0.28 ± 0.10	0.05 ± 0.04
**cGMP**	2.10 ± 0.80	0.39 ± 0.20	0.10 ± 0.08

FF: Follicular fluid; pOF: periovulatory oviductal fluid; CM: secretions of the cumulus cells. Results are expressed as mean ± standard deviation (SD).

**Table 2:** Motility of spermatozoa selected by chemotaxis to reproductive biofluids in the chemotaxis system. The effective concentration of chemoattractants were added to well B of the chemotaxis system: Follicular fluid (FF), periovulatory oviductal fluid (pOF), secretions of the cumulus cells (CM), progesterone (P4) and a combination of chemoattractants (Σ2 = 1/3 of 0.25% FF + 1/3 of 0.25% pOF + 1/3 of 0.06% CM). Spermatozoa (20 × 10^6^ cells/mL) were added to well A, and the system was incubated for 20 min. Motility parameters of the spermatozoa recovered in well B were determined. A group in which spermatozoa were selected without chemoattractant was used as the control (C). Four replicates were performed.

	Mot (%)	MotPro (%)	VCL (µm/s)	VSL (µm/s)	VAP (µm/s)	LIN (%)	STR (%)	WOB (%)	ALH (µm)	BCF (Hz)
**C**	47.0 ± 8.0	21.0 ± 7.2	65.8 ± 8.1	44.8 ± 8.7	49.3 ±8.0	65.7 ± 7.0	87.5 ± 4.5	74.0 ± 4.3	1.8 ± 0.2	6.3 ± 0.7
**FF**	28.5 ± 3.2	12.0 ± 3.8	42.0 ± 5.6	29.8 ± 5.0	32.8 ± 4.9	70.0 ± 3.6	89.5 ± 2.0	77.5 ± 2.2	1.5 ± 0.2	6.7 ± 0.7
**pOF**	41.7 ± 5.3	20.8 ± 4.8	54.7 ± 3.7	39.3 ± 4.8	42.8 ± 4.3	70.5 ± 5.6	90.2 ± 2.9	77.2 ± 3.8	1.7 ± 0.2	7.0 ± 0.3
**CM**	45.2 ± 7.6	23.5 ± 6.9	52.3 ± 7.5	34.3 ± 6.2	39.2 ± 6.3	65.2 ± 3.7	86.8 ± 2.5	74.5 ± 2.3	1.3 ± 0.2	6.5 ± 0.4
**P4**	37.5 ± 9.4	22.7 ± 0.4	53.8 ± 9.9	37.2 ± 8.9	41.0 ± 9.0	66.3 ± 8.8	87.8 ± 4.4	73.7 ± 6.9	1.7 ± 0.3	7.0 ± 0.8
**Σ2**	29.8 ± 4.2	12.3 ± 4.9	51.7 ± 5.5	35.7 ± 4.7	38.7 ± 4.8	68.3 ± 3.9	90.3 ± 2.2	75.0 ± 2.9	1.5 ± 0.2	7.3 ± 0.3

Mot (%): percentage of total motile spermatozoa; MotPro (%): percentage of motile progressive spermatozoa; VCL (µm/s): curvilinear velocity; VSL (µm/s): straight-line velocity; VAP (µm/s): average path velocity; LIN (%): linearity of the curvilinear trajectory; STR (%): straightness; WOB (%): Wobble (VAP/VCL); ALH (µm): amplitude of lateral head displacement; BCF (Hz): beat cross-frequency. Two-way ANOVA and multiple pairwise Tukey test (*p* < 0.05) was carried out. Results are expressed as mean ± SEM.

**Table 3:** In vitro fertilization (IVF) with spermatozoa selected by chemotaxis to reproductive biofluids in the chemotaxis system. The effective concentration of chemoattractants were added to well B of the chemotaxis system: Follicular fluid (FF), periovulatory oviductal fluid (pOF), secretions of the cumulus cells (CM) progesterone (P4), and a combination of chemoattractants (Σ2 = 1/3 of 0.25% FF + 1/3 of 0.25% pOF + 1/3 of 0.06% CM). Spermatozoa (20 × 10^6^ cells/mL) were added to well A, and the system was incubated for 20 min. A group in which spermatozoa were selected without chemoattractant was used as the control (C). Spermatozoa recovered in well B were used to perform the IVF at 25 × 10^3^ spermatozoa/mL. Eighteen hours after insemination, putative zygotes were fixed with glutaraldehyde and stained with the bisbenzimide solution. Evaluation was carried out by fluorescence microscopy. Four replicates were performed.

	*n*	Pen (%)	Mon (%)	Spz/O (*n*)	Spz/ZP (*n*)
C	126	40.5 ± 4.4 ^a^	33.3 ± 6.7	0.9 ± 0.1 ^a^	9.2 ± 0.9 ^a^
FF	139	66.2 ± 4.0 ^b^	34.0 ± 4.9	1.4 ± 0.1 ^b^	9.0 ± 0.5 ^a^
pOF	121	27.3 ± 4.1 ^a,c^	45.4 ± 8.8	0.5 ± 0.1 ^c,d^	2.6 ± 0.2 ^c,d^
CM	95	39.0 ± 5.0 ^a^	36.8 ± 7.9	0.7 ± 0.1 ^b,c^	5.3 ± 0.7 ^b^
P4	107	37.4 ± 4.7 ^a^	41.3 ± 8.0	0.7 ± 0.1 ^b,c^	4.8 ± 1.0 ^b,c^
Σ2	114	13.2 ± 3.2 ^c^	60.0 ± 13.1	0.2 ± 0.1 ^d^	1.2 ± 0.2 ^d^

Pen (%): percentage of oocytes penetrated; Mon (%): percentage of oocytes penetrate by one spermatozoon; Spz/O: mean number of spermatozoa penetrating one oocyte; Spz/ZP: mean number of spermatozoa bound to the zona pellucida. One-way ANOVA was performed and the Tukey test of multiple comparisons. Results are expressed as the mean ± SEM. Different superscript (^a–d^) within the same column indicate significant differences between experimental groups (*p* < 0.05).

**Table 4:** In vitro embryo development (IVC) of putative zygotes produced by in vitro fertilization (IVF) with spermatozoa selected by follicular fluid (FF) in the chemotaxis system. IVF was performed with 25 × 10^3^ selected spermatozoa/mL. After fertilization, putative zygotes continued the incubation in IVC medium (NCSU23) for up to 7 days (168 h). The percentage of embryos reaching 2-cells stage (48 h after insemination) and blastocyst and expanded blastocyst stages were analyzed (at 168 h after insemination). A group in which the spermatozoa were selected without chemoattractant was used as the control (C). Embryos after 7 days of culture were fixed with glutaraldehyde and stained with bisbenzimide solution. Evaluation was carried out by fluorescence microscopy. As an indicator of embryo quality, the number of cells forming each blastocyst was determined. Six replicates were performed.

	*n*	2-Cells (%)	Blastocyst		Expanded Blastocyst	
			%	Num. Cells	%	Num. Cells
C	824	42.1 ± 7.4	27.6 ± 3.8 ^a^	35.9 ± 2.4 ^a^	27.0 ± 2.2 ^a^	49.6 ± 2.1 ^a^
FF	718	39.8 ± 7.5	38.5 ± 3.7 ^b^	40.4 ± 2.3 ^b^	36.0 ± 2.5 ^b^	54.5 ± 2.4 ^b^

2-cells (%): percentage of oocytes that cleaved to the 2-cell stage of embryo development were evaluated at 48 h after insemination. Blastocyst and expanded blastocysts: percentage of 2-cells embryos that developed to blastocyst or expanded blastocyst stage (according to the classification proposed by Bó and Mapleroft, [26]) after 7 days (168 h) of culture. The number of cells were determined by fluorescence microscopy in blastocyst and expanded blastocysts fixed in 0.5% glutaraldehyde and stained with bisbenzimide solution. One-way ANOVA was performed and the Tukey test of multiple comparisons. Results are expressed as the mean ± SEM. The different superscripts (^a–b^) within the same column indicate the significant differences between groups (*p* < 0.05).

## Data Availability

The data presented in this study are available within the article or supplementary material.

## Supplementary Materials

The following are available online at https://www.mdpi.com/2076-2615/11/1/53/s1, Figure S1: Chemotaxis chamber diagram. Figure S2: Chemoattractant potential of chemoattractant combinations. Figure S3: Effect of CatSper inhibition on the membrane integrity of spermatozoa. Table S1: Effect of CatSper inhibition on the motility of spermatozoa.
