# Supplementary material for: Selection of Boar Sperm by Reproductive Biofluids as Chemoattractants
Source: Animals (Basel). 2020 Dec 30;11(1):53. doi: 10.3390/ani11010053 (PMC7824399; doi:10.3390/ani11010053)
Supplement: Supplementary file 1 [file animals-11-00053-s001.zip › Table S1.docx]

**Table S1. Effect of CatSper inhibition on the motility of spermatozoa.** Spermatozoa samples were preincubated for 10 min with 2 µΜ NNC 55-0396 (NNC) in TALP medium. Preincubated spermatozoa (20 x 10^6^ cells/mL) were added to well A, and the chemotactic system was incubated for 20 min. Motility parameters of the spermatozoa recovered in well B were determined with the CASA system. Preincubation with no supplements (C) and with the NNC solvent (DMSO) were used as controls. Three replicates were performed.

|  | Mot  (%) | MotPro  (%) | VCL  (µm/s) | VSL  (µm/s) | VAP  (µm/s) | LIN  (%) | STR  (%) | WOB  (%) | ALH  (µm) | BCF  (Hz) |
| --- | --- | --- | --- | --- | --- | --- | --- | --- | --- | --- |
| C | 33.0 ± 10.0 | 21.7 ± 6.7 | 50.0 ± 10.4 | 37.7 ± 9.0 | 41.3 ± 9.2 | 74.0 ± 5.3 | 90.7 ± 2.6 | 81.0 ± 3.6 | 1.0 ± 0.0 | 6.7 ± 0.9 |
| NNC | 15.0 ± 2.9 | 9.3 ± 1.8 | 35.3 ± 8.2 | 21.3 ± 6.4 | 25.3 ± 6.3 | 60.0 ± 8.5 | 82.7 ± 4.4 | 71.7 ± 7.1 | 1.0 ± 0.0 | 5.0 ± 0.6 |
| DMSO | 36.3 ± 8.6 | 23.3 ± 3.8 | 50.0 ± 7.5 | 35.3 ± 5.7 | 41.3 ± 5.5 | 71.0 ± 4.6 | 85.7 ± 4.0 | 82.3 ± 1.4 | 1.3 ± 0.3 | 6.0 ± 0.6 |

Mot (%): percentage of total motile spermatozoa; MotPro (%): percentage of motile progressive spermatozoa; VCL (µm/s): curvilinear velocity; VSL (µm/s): straight-line velocity; VAP (µm/s): average path velocity; LIN (%): linearity of the curvilinear trajectory; STR (%): straightness; WOB (%): Wobble (VAP/VCL); ALH (µm): amplitude of lateral head displacement; BCF (Hz): beat cross-frequency. Two-way ANOVA and multiple pairwise Tukey test (p<0.05) was carried out. Results are expressed as mean ± SEM.
