# Supplementary figures and images for: Selection of Boar Sperm by Reproductive Biofluids as Chemoattractants
Source: Animals (Basel). 2020 Dec 30;11(1):53. doi: 10.3390/ani11010053 (PMC7824399; doi:10.3390/ani11010053)

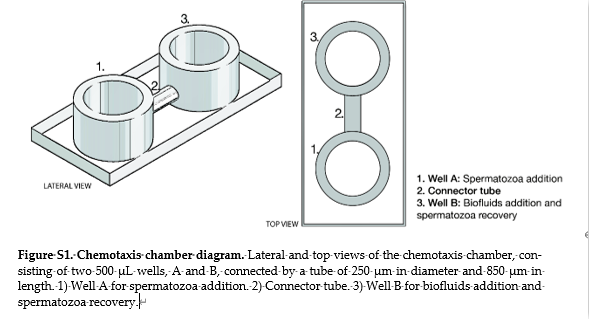

Supplement: Supplementary file 1 [file animals-11-00053-s001.zip › Fig S1.PNG]

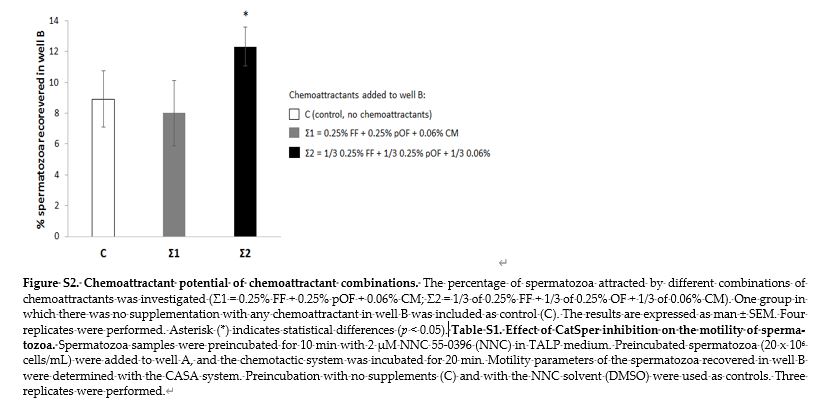

Supplement: Supplementary file 1 [file animals-11-00053-s001.zip › Fig S2.JPG]

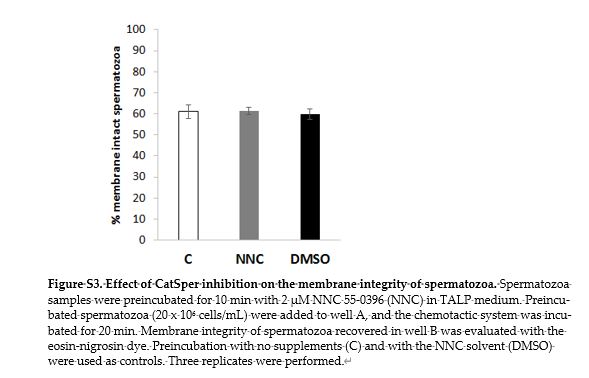

Supplement: Supplementary file 1 [file animals-11-00053-s001.zip › Fig S3.JPG]
